# How p53 Molecules Solve the Target DNA Search Problem: A Review

**DOI:** 10.3390/ijms21031031

**Published:** 2020-02-04

**Authors:** Kiyoto Kamagata, Yuji Itoh, Dwiky Rendra Graha Subekti

**Affiliations:** 1 Institute of Multidisciplinary Research for Advanced Materials, Tohoku University, Katahira 2-1-1, Aoba-ku, Sendai 980-8577, Japan; yuji_itoh@nig.ac.jp (Y.I.); Dwikyren@dc.tohoku.ac.jp (D.R.G.S.); 2 Department of Chemistry, Graduate School of Science, Tohoku University, Sendai 980-8578, Japan; 3 Genome Dynamics Laboratory, National Institute of Genetics, Mishima, Shizuoka 411-8540, Japan

**Keywords:** p53, DNA, single molecule fluorescence microscopy, sliding, diffusion, intersegmental transfer, target search

## Abstract

Interactions between DNA and DNA-binding proteins play an important role in many essential cellular processes. A key function of the DNA-binding protein p53 is to search for and bind to target sites incorporated in genomic DNA, which triggers transcriptional regulation. How do p53 molecules achieve “rapid” and “accurate” target search in living cells? The search dynamics of p53 were expected to include 3D diffusion in solution, 1D diffusion along DNA, and intersegmental transfer between two different DNA strands. Single-molecule fluorescence microscopy enabled the tracking of p53 molecules on DNA and the characterization of these dynamics quantitatively. Recent intensive single-molecule studies of p53 succeeded in revealing each of these search dynamics. Here, we review these studies and discuss the target search mechanisms of p53.

## 1. Introduction

Genome editing method has been used to investigate and engineer gene functions in life science. The genome encompasses DNA sequences that encode genes, and gene editing is the genetic engineering of a specific DNA sequence, including insertion, deletion, modification, and replacement. The main player in genome editing is a type of protein that can bind to DNA, known as DNA-binding proteins. DNA-binding proteins include enzymes, which can cut DNA or ligate two DNA molecules, and transcription factors, which can activate or deactivate gene expression. These proteins are classified into DNA sequence-specific and nonspecific binders.

The sequence-specific DNA-binding proteins have a common mechanism to search for and bind to their target DNA sites encoded in a genome. Successful target binding by transcription factors can trigger the regulation of the cellular functions, whereas the failure of the target search and binding is known to cause various diseases including cancers. In addition, the target binding is delicately regulated depending on genome functioning. Accordingly, the target search and binding by DNA-binding protein are one of the indispensable reactions in life. The bacterial and human genomes comprise approximately 10^6^ and 10^9^ bp of DNA, respectively. In contrast, the DNA sequence that is bound by the proteins is just typically 5–30 bp. For example, assuming that a single protein searches for the target coded in 10^9^ bp of genome by repeatedly associating and dissociating with random sites in the genome (3D diffusion), the search time is estimated to be much longer than the physiological time which is estimated from several minutes to a half of an hour in cells. However, the natural proteins complete the target search exactly within the physiological search time. This gap is called the “target search problem.” How do DNA-binding proteins solve this problem and function on the target?

To solve this problem, three target search dynamics are currently proposed: 3D diffusion; 1D diffusion, in which a protein moves along DNA; and intersegmental transfer, in which a protein transfers between two different DNA strands without dissociating [1,2,3]. Theoretical studies demonstrate that a combination of at least two dynamics can facilitate the target search [1,2,4,5], known as facilitated diffusion.

## 2. p53 As a Model System for Target Search Studies

**Figure 1:**
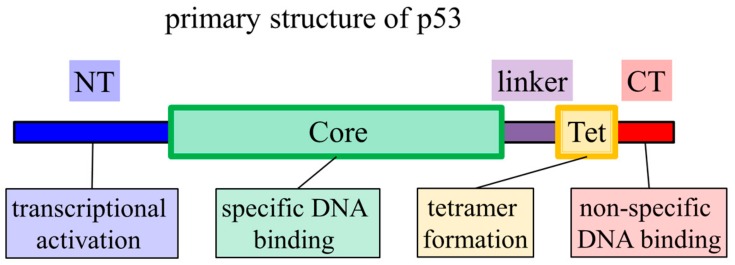
Domain organization and function of p53. Thin and thick boxes represent disordered and folded regions, respectively. NT, core, Tet, and CT function as transcriptional regulation, target DNA sequence binding, dimer or tetramer formation, and non-target DNA sequence binding, respectively.

The transcription factor p53 can induce multiple tumor suppression functions, such as cell cycle arrest, DNA repair, and apoptosis. Furthermore, p53 is known as the guardian of the genome, and it contains an N-terminal (NT) domain, core domain, linker, tetramerization (Tet) domain, and C-terminal (CT) domain (Figure 1). The core and Tet domains have specific folded structures, while the others are intrinsically disordered regions [6,7,8]. The core and CT domains are involved in binding to DNA sequences in a specific and nonspecific manner, respectively [9]. The Tet domain enables the formation of a homo-tetramer [8]. In target search study, p53 is used as a model protein because it has common features that are frequently observed in DNA-binding proteins, such as oligomerization, a disordered region, and multiple DNA-binding domains [10]. A defect in target binding by p53 causes tumorigenesis [11], indicating the importance of successful target searching and binding.

## 3. Single-Molecule Fluorescence Microscopy

**Figure 2:**
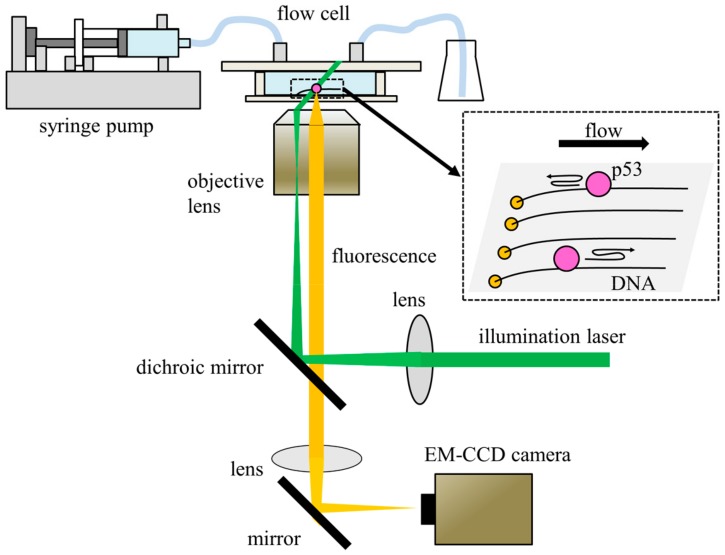
Schematic diagram of representative single-molecule fluorescence microscopy. The system comprises a fluorescence microscope and a flow cell. Fluorescently labeled p53 is introduced into the flow cell using a syringe pump. DNA is tethered on one end to the MPC-polymer-coated glass surface of the flow cell in a line using the DNA garden method. The tethered DNA molecules are stretched by flow pressure. TIRF or HILO is used to illuminate DNA-bound p53 molecules. Fluorescence from the p53 molecules is detected by EM-CCD. The figure is adapted from ref. [25] with some modifications.

To solve the target search problem, it is necessary to differentiate and characterize each search dynamic. Single-molecule fluorescence microscopy can track a single molecule moving along DNA and/or transferring between two DNA molecules [12,13,14,15,16,17,18,19,20,21,22,23,24]. In general, the system contains a fluorescence microscope and a flow cell (Figure 2). Fluorescent dye-labeled p53 is introduced into the flow cell using a syringe pump. At least one end of each DNA molecule is tethered to the inner glass surface of the flow cell, and the tethered DNA is stretched by the flow pressure. The molecules, which are bound to the DNA, are detected as fluorescent spots using an electron-multiplying charge-coupled device (EM-CCD) camera of a fluorescence microscope. Total internal reflection fluorescence (TIRF) or highly inclined and laminated optical sheet (HILO) is used to illuminate DNA-bound molecules selectively and to reduce the unexpected background fluorescence. The positions of individual molecules are tracked using an appropriate analysis program.

Several elegant methods for producing DNA arrays were developed in order to acquire data on many molecules simultaneously [26,27,28]. A simple method is the “DNA garden,” in which neutravidin molecules are printed in a line on coverslips using polydimethylsiloxane (PDMS) stamps, and then biotinylated DNAs are tethered to the stamped neutravidin [28] (Figure 2). In addition, 2-methacryloyloxyethyl phosphorylcholine (MPC) polymer coating minimizes the adsorption of p53 and DNA to the flow cell, which distorts measurements. The DNA array can reduce the overlap of two adjacent DNA molecules and simplify analysis.

## 4. 1D Diffusion of p53 along DNA

In 2008, Tafvizi et al. succeeded in observing the movement of p53 tetramer along a non-target DNA sequence using single-molecule fluorescence microscopy [29]. A thermostable mutant of human p53 was used as pseudo-wild type to prevent irreversible aggregation observed in genotypic wild type in some cases. Also, we consider only the tetrameric form of full-length p53 throughout the manuscript because of the main oligomeric state for the target search (see in detail in Section 6). The single-molecule tracking revealed that p53 diffuses forward and backward along DNA randomly. The 1D diffusion is caused only by the thermal energy, but not the chemical energy of ATP. In other words, p53 converts the thermal energy to drive 1D search dynamics along DNA by maintaining weak interactions between p53 and DNA. Before this study, McKinney et al. indirectly observed p53 sliding off from short DNAs in ensemble gel shift assay, thereby suggesting 1D diffusion by p53 [30]. The 1D diffusion of p53 was later confirmed using similar single-molecule fluorescence microscopy by our group [31] as well as course-grained molecular dynamic (MD) simulations by two groups [32,33]. Accordingly, single-molecule tracking provided direct evidence of 1D diffusion by p53 along DNA.

Tafvizi et al. further revealed that 1D diffusion of p53 along the non-target DNA sequence does not depend on a monovalent salt concentration; this suggested “1D sliding,” in which p53 moves along DNA while maintaining contact with the DNA [2,29]. Based on similar measurements of p53 mutants with deletions in either of the DNA-binding domains, it was proposed that p53 searches for the target using restricted hopping of the core domains during 1D sliding [34]. This was later supported by a course-grained MD simulation [33]. Leith et al. also found that 1D diffusion of p53 slowed on DNA sequences that were definitely non-target sequences but had higher similarity to the target sequence [35]. This finding suggests that p53 that diffuses along DNA can read the DNA sequence through interactions between its core domains and DNA.

**Figure 3:**
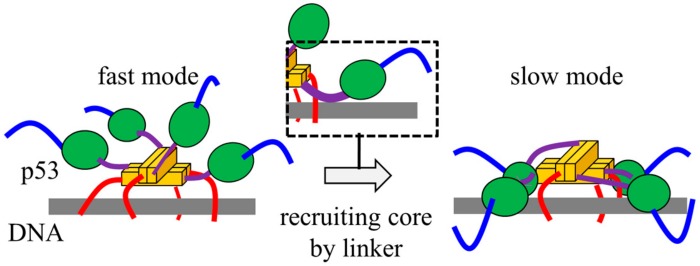
Schematic diagram of the p53-DNA complex structure in 1D diffusion along a non-target DNA sequence. The linker (purple) recruits the core domain (green) to DNA (grey), triggering the conformational switch between fast and slow 1D diffusion modes. The color of p53 domains is the same in Figure 1.

We clarified that p53 possesses two 1D diffusion modes with different diffusion coefficients by altering the structure of p53 on DNA [31,36] (Figure 3). The fast 1D diffusion mode enables an extension of the search distance by maintaining weak contacts between the CT domains and DNA. In the slow diffusion mode, p53 has a similar conformation that can recognize and bind to the target DNA sequence on non-target DNA sequence. In particular, the core domains as well as CT domains participate in p53 binding to DNA. Furthermore, the disordered linker connecting the core and Tet domains can trigger switching between the two 1D diffusion modes via electrostatic interactions between the linker and DNA [37] (Figure 3). Overall, p53 performs the target search using 1D diffusion coupled with flexible conformational changes on DNA.

If p53 reaches the target DNA sequence using 1D diffusion, it will recognize and bind to the target. Considering the fast target search by DNA-binding proteins, one might predict a high efficiency of target recognition and binding [38]. Contrary to this prediction, we revealed that only ~10% of p53 molecules succeed in binding to the target DNA sequence [39]. Surprisingly, ~90% of molecules pass over the target sequence without recognition. This low probability of target recognition might be explained by a large conformational change by p53 on the target DNA sequence, as predicted in course-grained MD simulations [40]: p53 was unable to achieve the target-binding conformation before diffusing away from the target. Accordingly, the target search by p53 is decelerated due to the low target recognition probability, although 1D diffusion contributes to facilitating the target search. On the other hand, p53 function can be regulated by altering the target recognition probability in response to the cell condition [39]. Interestingly, evolution selects regulation of the target recognition probability rather than target search facilitation.

## 5. Ultrafast Intersegmental Transfer of p53

**Figure 4:**
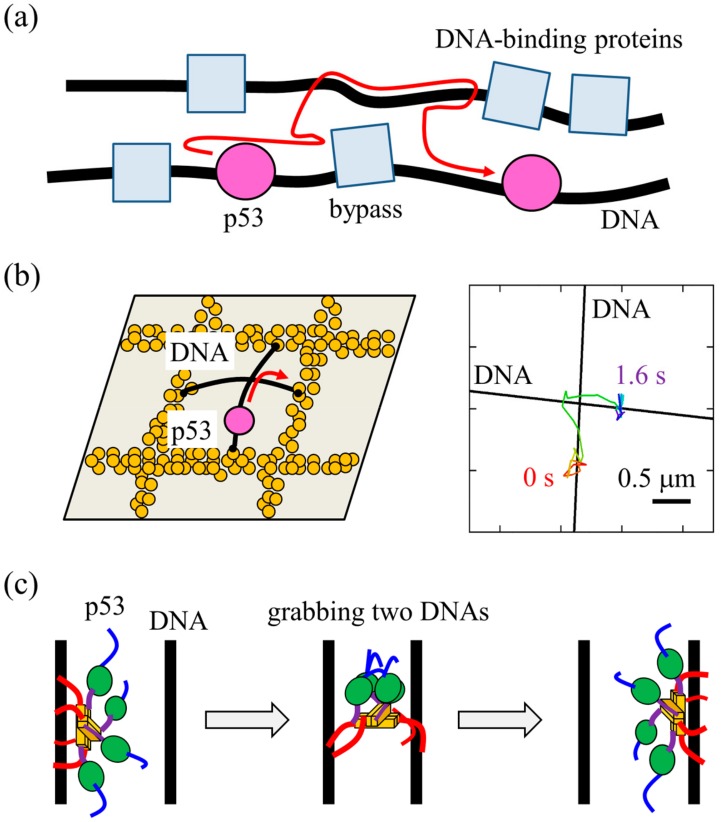
(**a**) Schematic diagram of target search by p53 in the cell. p53 (pink spheres) may bypass other DNA-binding proteins (light blue squares) bound to DNA (black lines) using intersegmental transfer. (**b**) Single-molecule verification of intersegmental transfer of p53 between crisscrossing DNAs (left). The typical data of p53 demonstrating intersegmental transfer (right). (**c**) Schematic diagram of intersegmental transfer of p53. p53 binds to the first DNA mainly using the CT domain (Left), grabs the second DNA (Middle), and then releases the former while maintaining contact with the latter DNA (Right). The panels (**a**) and (**c**) are adapted from ref. [25] with some modifications, and the panel (**b**) is adapted from ref. [41] with some modifications.

In the cell, many DNA-binding proteins bind to and cover DNA, which could block the 1D diffusion of p53 along DNA and hinder its target search. In the nucleus, genomic DNA is stored at high concentrations. In these conditions, it may be possible that p53 possesses a special mechanism to bypass the obstacles on DNA by transferring from one DNA strand to another (Figure 4a).

We conducted a kinetic ensemble measurement to monitor the transfer process of p53 from fluorescent to non-fluorescent DNA [41]. Fluorescence anisotropy was used to differentiate between the p53-DNA complex and free DNA by monitoring rotational DNA diffusion. After p53 bound to the fluorescent DNA was mixed with a large quantity of non-fluorescent DNA, p53 transfer to the non-fluorescent DNA was observed. The data demonstrated that p53 transfers between two non-target DNA strands at nearly the diffusion limit (~10^8^ M^−1^s^−1^). This suggests that even if p53 encounters DNA-bound obstacles in the cell, it can bypass these via ultrafast transfer. In contrast, p53 does not transfer from the target DNA sequence. Accordingly, once p53 binds to the target DNA sequence, it is not repelled from the target DNA by nearby DNA in the cell.

To directly examine p53 transfer between DNA segments, we prepared crisscrossing DNA in the flow cell using the modified DNA garden method and tested whether p53 moves between DNA segments at the crisscross points [41] (Figure 4b). The crisscrossing DNA was prepared according to the method developed by Greene’s group [42]. Notably, p53 changed the direction of 1D diffusion at an ~90 degree angle at a crisscross point, which gave direct evidence of intersegmental transfer. Further analysis of the mutants lacking either of the DNA-binding domains demonstrated that CT domains are required for intersegmental transfer. Therefore, during intersegmental transfer, p53 bound to the first DNA should grab the second DNA using at least two of four CT domains, after which it releases the first and transfers to the second DNA (Figure 4c). These experimental data are consistent with the preceding results based on course-grained MD simulations by the Levy and Takada groups [10,43].

## 6. Target Search by p53 May Be Well Designed by Biological Requirements

Considering the in vitro data described above, p53 is presumed to solve the target search problem by utilizing 3D diffusion, 1D diffusion along DNA, and intersegmental transfer between two DNAs in the cell. In 3D search, p53 can associate to non-target DNA near the diffusion limit [39]. The association rate constant of p53 is 1–10000-fold faster than those of other DNA-binding proteins including transcription factors and a nucleoid protein [44,45,46,47,48,49,50,51,52,53,54,55,56,57,58]. This ultrafast association enables a reduction in the time taken for 3D diffusion in which p53 cannot search the target in solution. The 1D diffusion enables p53 to search ~200 bp of DNA before dissociating [36] and contributes to ~10-fold elongation of the search distance compared to that in the absence of 1D diffusion. The intersegmental transfer of p53 occurs 10–10,000-fold faster than that of other DNA-binding proteins [59,60,61,62,63,64], with reactions near the diffusion limit [41]. Using such ultrafast intersegmental transfer, p53 could bypass DNA-bound obstacles in the cell, thereby facilitating the target search. In contrast, as described above, the target recognition efficiency of p53 was surprisingly low. These findings indicate that not all target searches by p53 are necessarily optimized toward facilitation.

Considering the biological functions of p53, one may wonder why the target search by p53 is not optimized in terms of facilitation. Under various cellular stresses such as DNA damage and tumorigenesis of the cell, p53 turns the function “ON” and induces DNA repair and apoptosis. In contrast, p53 should keep the function “OFF” under normal conditions. It would be dangerous if p53 were to accidentally bind to the target DNA sequences and induce apoptosis in normal conditions. To prevent this malfunction, p53 may keep target recognition low under normal conditions at the expense of target search speed in stress conditions.

To regulate multiple functional pathways as well as the discrete ON/OFF switch, p53 has three different strategies. One strategy is to increase the p53 copy number under stressed conditions [65]. The increased copy number itself can promote target searching and binding, thus triggering its function. In addition, further p53 expression enables lower affinity binding to the target DNA sequences, thereby switching the functional pathways [66]. Concomitant with the increase in copy number, the oligomeric state of p53 is altered [67,68,69], as the major oligomeric state changes from dimer to tetramer upon cell stress [70]. The affinity of the p53 dimer to the target DNA sequence is known to be much weaker than that of the tetramer [71]. Accordingly, the tetramer is the main oligomeric state involved in the target search and can regulate function. The other functional regulator is posttranslational modification [72,73,74,75,76,77]. Phosphorylation of the CT domain enhances target recognition, thereby enhancing the transcriptional activity [38,78]. Acetylation of the CT domain modulates target binding in vitro and in the cell [79,80]. Overall, these multiple strategies may be required to activate only the appropriate functional pathway according to cell conditions.

## 7. Target Search of p53 in Live Cells

The target search dynamics of p53 in vivo have been investigated by single-molecule fluorescence imaging. For example, Mazza et al. succeeded in tracking p53 molecules one by one in live cells using HILO illumination [81]. They showed that 20% of p53 molecules bound to chromatin and the other molecules diffused freely in the nucleus. Furthermore, Morisaki et al. found that the p53 molecules have two residence times (0.33 s and 3.5 s) for binding to transcription domains of cells, whereas the long residence time was not detected outside of the transcription domains [82]. Based on mutational analysis, they identified that the long residence time corresponds to the binding to the target sites. The percentage of the successful target binding was estimated to be 5.5% within the transcription domains. In contrast, the short residence time represents a mixture of slowly diffusing and/or transiently bound molecules. During the transient binding to genomic DNA, p53 could search for the target sites by sliding along DNA and/or using intersegmental transfer. Considering that p53 molecules search for the target sites out of the transcription domains, the percentage of the successful target binding in vivo would be quite low. Interestingly, the residence times of p53 on target and non-target DNAs in vivo are much longer than those obtained by in vitro measurements [39]. The molecular crowding effect or proteins bound to p53 may stabilize the p53/DNA complex in cells. In addition, the post-translational modifications to p53 can modulate the residence time on target sites, which enhances the transcriptional activity [80]. Further investigations are required to clarify the behavior of p53 in cells.

## 8. Common Target Search in Biology

Target search is not limited to DNA-binding proteins, such as p53. Various processes can be considered as target search problems, including biomolecules searching for binding partner molecules or animals seeking food. Interestingly, a common mechanism known as intermittent search is widely used in biology [38]. Intermittent search involves two different modes: a recognition mode, in which molecules can recognize their objectives, and a search mode, in which molecules can cover long distances without target recognition. In the case of p53, the search and recognition modes correspond to 3D diffusion (and/or intersegmental transfer) and 1D diffusion along DNA, respectively. Furthermore, 1D diffusion also comprises the two similar modes, thereby facilitating the target search. Accordingly, p53 utilizes the common intermittent search. It is interesting that biomolecules and living things on the micro-to-macro scale select the common search strategy in time scales from seconds to hours.

## 9. Summary and Future Perspectives

Single-molecule fluorescence microscopy has clarified that p53 solves the target search problem by utilizing 3D diffusion, 1D diffusion along DNA, and intersegmental transfer between two DNAs. As predicted by theoretical studies, target search by p53 can be facilitated by combining these three strategies. In particular, considering the ultrafast association to DNA, 1D diffusion along DNA, and ultrafast intersegmental transfer, target searching by p53 is somehow optimized toward facilitation. In contrast, the low target recognition by p53 decelerates target searching. Furthermore, p53 may prevent malfunction under normal conditions at the expense of the target search speed in stress conditions. Accordingly, the target search strategy of p53 may be well designed to satisfy biological requirements.

Further single-molecule investigations could deepen the understanding of target search by p53. In the cell, histone proteins bind to and condense DNA, which may affect target search by p53 [83]. Single-molecule measurements with the use of histone-bound DNA [84], an in vivo-mimicking system used in vitro would offer further interpretation of the in vivo single-molecule data [80,81,82]. Also, single-molecule microscopy could be used to validate important DNA binding regions as well as local DNA structures of p53 [85,86,87,88,89,90] and to clarify the molecular mechanism for functional regulations of truncated and/or posttranslational modified forms of p53 [91,92,93,94,95]. Additionally, p53 may search for the target using hops and short jumps along DNA, as observed in EcoRV [96]. To resolve these dynamics, the spatial and/or temporal resolutions of the single-molecule microscope must be improved. Recently, we succeeded in improving such temporal resolution from 33 ms to 0.5 ms and found that p53 hops and jumps along DNA (Subekti et al., submitted). Furthermore, cryo-electron microscopy could determine heterogeneous structures of p53 at the single-molecule level [97,98,99]. In addition to the single-molecule measurements, the regulation of the target search dynamics as well as target binding by an artificially designed peptide may be useful for medical applications [100].

Since many DNA-binding proteins exist in the cell and have different structures and functions, it is interesting how such DNA-binding proteins solve the target search problem. For example, if certain DNA-binding proteins differ in structure and function, their target search mechanisms may each differ as well [101,102,103,104,105]. In the near future, the target search mechanism of various DNA-binding proteins will be revealed by single-molecule fluorescence microscopy. Comparisons between p53 and other proteins will allow the characterization of the special action of p53 in performing target search. Furthermore, such detailed characterization could give us suggestions for engineering of gene-editing DNA-binding proteins toward higher editing efficiency and lower off-target editing.

## Abbreviations

NT	N-terminal
Tet	tetramerization
CT	C-terminal
TIRF	total internal reflection fluorescence
HILO	highly inclined and laminated optical sheet
PDMS	polydimethylsiloxane
MPC	2-methacryloyloxyethyl phosphorylcholine
MD	molecular dynamics
